# Evaluating progestogens for prevention of preterm birth international collaborative (EPPPIC) individual participant data (IPD) meta-analysis: protocol

**DOI:** 10.1186/s13643-017-0600-x

**Published:** 2017-11-28

**Authors:** Lesley A. Stewart, Mark Simmonds, Lelia Duley, Kristina Charlotte Dietz, Melissa Harden, Alex Hodkinson, Alexis Llewellyn, Sahar Sharif, Ruth Walker, Kath Wright, the EPPPIC group

**Affiliations:** 1 Centre for Reviews and Dissemination, University of York, Heslington, York, YO10 5DD UK; 2 Nottingham Clinical Trials Unit Queen’s Medical Centre, University of Nottingham, Nottingham, NG7 2UH UK

**Keywords:** Preterm birth, Progestogen, Individual participant data, IPD, Meta-analysis

## Abstract

**Background:**

Preterm birth is the most common cause of death and harm to newborn babies. Babies that are born early may have difficulties at birth and experience health problems during early childhood. Despite extensive study, there is still uncertainty about the effectiveness of progestogen (medications that are similar to the natural hormone progesterone) in preventing or delaying preterm birth, and in improving birth outcomes. The Evaluating Progestogen for Prevention of Preterm birth International Collaborative (EPPPIC) project aims to reduce uncertainty about the specific conditions in which progestogen may (or may not) be effective in preventing or delaying preterm birth and improving birth outcomes.

**Methods:**

The design of the study involves international collaborative individual participant data meta-analysis comprising systematic review, re-analysis, and synthesis of trial datasets.

Inclusion criteria are as follows: randomized controlled trials comparing progestogen versus placebo or non-intervention, or comparing different types of progestogen, in asymptomatic women at risk of preterm birth. Main outcomes are as follows; fetal/infant death, preterm birth or fetal death (<=37 weeks, <=34 weeks, <= 28 weeks), serious neonatal complications or fetal/infant death, neurosensory disability (measured at 18 months or later) or infant/child death, important maternal morbidity, or maternal death. In statistical methods, IPD will be synthesized across trials using meta-analysis. Both ‘two-stage’ models (where effect estimates are calculated for each trial and subsequently pooled in a meta-analysis) and ‘one-stage’ models (where all IPD from all trials are analyzed in one step, while accounting for the clustering of participants within trials) will be used. If sufficient suitable data are available, a network meta-analysis will compare all types of progesterone and routes of administration extending the one-stage models to include multiple treatment arms.

**Discussion:**

EPPPIC is an international collaborative project being conducted by the forming EPPPIC group, which includes trial investigators, an international secretariat, and the research project team. Results, which are intended to contribute to improvements in maternal and child health, are expected to be publicly available in mid 2018.

**Systematic review registration:**

PROSPERO CRD42017068299

## Plain language summary

Preterm birth is the most common cause of death and harm to newborn babies. In the USA, about one in every 10 babies is born preterm, before the 37th week of pregnancy. Preterm birth is more common in African American women, affecting about one in every seven babies. In Europe, about 1 in 20 births is preterm, and in African countries, almost 1 in 5 babies are delivered before 37 weeks.

Babies born early may have difficulties at birth and health problems during early childhood. They are more likely to die during their first year and to experience long-term disabilities such as cerebral palsy, epilepsy, blindness, and hearing loss. Reducing the numbers of preterm births is therefore important to pregnant women and their families, and is a global health priority.

Progestogens are medications that are similar to the pregnancy hormone progesterone. They are thought to help prevent preterm birth in women who may be at higher risk of an early birth. Women who have had a preterm birth in a previous pregnancy, have short cervical length, or are carrying twins or triplets are at greater risk of having a preterm birth. There are different types of progestogen. Some are given as weekly injections (shots). Others are given as daily vaginal gels or tablets during the second and third trimesters of pregnancy.

In total, around 45 clinical trials have studied the use of progestogen in these types of pregnancies. Many of these studies have been small and results have been mixed. Several reviews of these trials have also been completed. Some reviews have suggested that progestogen reduces the rate of preterm birth, while others have found no evidence of benefit. Further trials have been completed since these reviews were published.

The Evaluating Progestogens for Prevention of Preterm birth International Collaborative (EPPPIC) has been set up to reduce uncertainty about the specific conditions in which progestogen may (or may not) be effective in preventing or delaying preterm birth and improving birth outcomes. It will help us learn to whom it should be offered and in what form. EPPPIC aims to identify all relevant clinical trials and to collect and analyze the data collected on every woman participating in these trials. It will combine similar data across multiple studies. This is known as an individual participant data (IPD) meta-analysis. Having access to the ‘raw’ data collected in the trials will allow the research team to carry out very detailed analysis and better understand how and when progestogen may help.

The data will be used to compare use of progestogens to standard obstetric care (with no progestogens) and to compare different types of progestogen. The effect of progestogens on preventing preterm birth, infant death, complications at birth, and long-term disability will be investigated. Of particular interest will be the effect of progestogens in women with a twin (or triplet) pregnancy, in women who have had a preterm birth in a previous pregnancy, and in women with a short cervix (which increases the risk of preterm birth).

This work is being carried out by a research team based at the University of York, in partnership with individual trial investigators and with a secretariat comprising funders, and key trial and stakeholder representatives. Findings will be published by the EPPPIC group.

## Introduction

### Background

Preterm birth (defined as birth before 37 weeks of gestation) is the most common cause of neonatal morbidity and mortality globally, with rates ranging from 5% in European countries to 18% in African countries [1]. In the USA, 9.84% of all babies and 13.75% of those born to non-Hispanic black mothers were delivered preterm in 2016 (https://www.cdc.gov/nchs/data/vsrr/report002.pdf).

Infants born prematurely, particularly those born before 34 weeks, are at greater risk of having difficulties at birth and health problems during infancy including greater risks of dying during their first year [2]. They are also more likely to experience long-term morbidity such as cerebral palsy, epilepsy, blindness or hearing loss. Preterm birth can therefore have lifelong impact for individuals and families. It can also have important economic consequences, for families as well as for payers and purchasers of health care. In 2005, the Institute of Medicine estimated the annual societal cost of preterm birth in the USA to be $26 billion [3]. Reducing rates of preterm birth would therefore accrue significant health and fiscal benefits.

Endogenous progesterone is important in maintaining pregnancy, and decline of progesterone activity is believed to play a role in the onset of labor. Progestogens (compounds with progesterone-like action) have been regarded as promising therapeutic agents since the 1960s. Natural progestogens are similar to those produced by living organisms, while synthetic progestogens including 17-hydroxyprogesterone caproate (17-OHPC) are manufactured in the laboratory and have a different chemical structure [4].

The cost of licensed proprietary 17-OHPC in 2011/2012 has been reported as $690 per injection or around $15,000 per pregnancy [5, 6], the cost of compounded 17-OHPC as $285 per pregnancy, and vaginal gels and suppositories as between $338 and $2499 per pregnancy [5].

### Current evidence and knowledge

The role of progestogen (mainly vaginal administration of progesterone and intramuscular injection of 17-OHPC) in preventing preterm birth has been extensively studied in over 40 randomized controlled trials (RCTs) and in several systematic reviews.

A Cochrane review of 36 RCTs, including 8523 women and 12,515 infants [7] found that progestogens administered by intramuscular injection or vaginal application prolonged pregnancy and reduced neonatal mortality and short-term morbidity. It noted that there was little information on longer-term outcomes.

An IPD-MA that focused on vaginal administration of progesterone in women with short cervix in mid-trimester (< 25 mm), and included data from 5 RCTs, 775 women and 827 infants [8] provided strong evidence in favor of intervention. However, a 2015 IPD-MA examining effects in twin pregnancies that included 13 trials, 3768 women and 7536 babies found that 17-OHPC did not reduce the incidence of a composite outcome of adverse perinatal outcomes that included preterm birth; though a subgroup analysis suggested some benefit in women with reduced cervical length [9]. An IPD-MA of 3 RCTs of 232 triplet pregnancies found no significant impact of 17-OHPC on outcome or duration of pregnancy [10].

A large placebo controlled randomized trial of vaginally administered progesterone that included 1228 women and focused on family-centered outcomes published in 2016 found no significant effect on the primary obstetric or neonatal outcomes, including longer-term child health at 2 years of age [11]. When the relevant subset of women enrolled in this trial were included in a meta-analysis of women with singleton pregnancies and a short cervix length [12], the combined evidence showed significant benefit of vaginal progesterone in reducing the risk of preterm birth and neonatal morbidity and mortality. An updated meta-analysis of twin gestation pregnancies in 303 women with a short cervix enrolled from 6 RCTs [13] found a decrease in the risk of preterm birth and some neonatal morbidities.

A recent systematic review included 680 women enrolled in 3 RCTs compared vaginal progesterone versus 17-OHPC in singleton pregnancies. However, as this used aggregate data extracted from trial publications, it was limited in scope, but reported similar effectiveness for vaginal progesterone and 17-OHPC injection [14].

### Rationale for a new systematic review with IPD meta-analysis

Despite extensive study, there still remains uncertainty regarding the specific conditions in which progestogen may (or may not) be effective in preventing or delaying preterm birth and in improving birth outcomes. Optimal dose, route, and formulation of progestogens have not been established. Resolving this uncertainty could have important impact and provide more robust evidence to help pregnant women at risk of preterm birth make informed choices. Given the lifelong consequences, even a small change in preterm birth rate could yield significant benefit.

A comprehensive and up-to-date IPD-MA of all randomized trials comparing progestogens with placebo or routine care is a potentially powerful approach to resolving current uncertainty about the effectiveness of progesterone in preventing preterm birth.

Although several completed systematic reviews exist, each has looked at different subsets of the at-risk population and several new trials have since been completed. The EPPPIC IPD-MA aims to bring together the totality of existing evidence from randomized trials. It will enable detailed analyses that can more appropriately account for heterogeneity within and between trials than analyses based on aggregate data, and will apply the same methods across different at risk populations. An IPD network meta-analysis that also includes trials that have compared vaginally administered progesterone, 17-OHPC, and oral progesterone will provide important additional evidence on comparative effectiveness and safety.

The IPD approach will support analyses to explore whether there are particular types of women or pregnancy who gain a greater degree of benefit from progestogens, or, conversely, women who gain little or no benefit and who may therefore prefer to avoid the potential side effects of intervention.

### Collaborative approach

EPPPIC will be an international partnership involving trial investigators who have carried out eligible randomized trials, members of the secretariat and the IPD-MA research team, who will work together to provide a definitive evaluation of the existing evidence. The EPPPIC IPD-MA research team, who have developed this protocol, have previously completed and published a number of such collaborative IPD meta-analyses, which are illustrative of the collaborative model [e.g., [15, 16]]. The EPPPIC secretariat includes two consumer representatives who will provide perspective and feedback throughout the project. We will also consult more widely with relevant professional stakeholders, charities, and support organizations in relation to the appropriateness of outcomes and dissemination activity.

### Aims and objectives

The aims of this study are to undertake a robust evaluation of the benefits and harms of progestogens in preventing preterm birth and associated morbidity and to determine to whom progestogen should be offered, and in what form. This will contribute to improved health and well-being of pregnant women and to improved child health.

This systematic review and IPD-MA will assess the benefits and harms of progestogen (in different forms, doses, routes of administration, and timing) administered for the prevention of preterm birth. It will investigate whether any particular therapeutic approaches are more effective than others, including evaluation of the comparative effectiveness of vaginal progesterone and 17-OHPC. It will evaluate:

1. Effectiveness of progestogen versus no active intervention
  (additional early co-treatment, e.g., cerclage, pessary; and/or late treatment, e.g. tocolytics, is allowed provided that permitted use does not differ between trial arms)
  Separate analyses will be carried out for each type of progestogen. Further *exploratory analyses may* combine types of progestogen if there is no evidence of differences in effectiveness between them.
2. Effectiveness of vaginally administered progesterone versus 17-OHPC versus oral progesterone
  (additional early co-treatment, e.g., cerclage, pessary; and/or late treatment, e.g. tocolytics, is allowed provided that permitted use does not differ between trial arms)

It will also explore whether there are particular types of women or pregnancy who derive greater benefit (or harm) from intervention. Key clinical scenarios that reflect patient populations defined by risk factor will be explored and emphasized. Emphasis will be placed on (but analyses not limited to) the following:

- Twin pregnancy with previous preterm birth and maternal short cervix
- Twin pregnancy with previous spontaneous preterm birth (any cervical length)
- Twin pregnancy with maternal short cervix, but no previous preterm birth
- Twin pregnancy with no other risk factors
- Triplet pregnancy (likely to be too few trial participants to split further)
- Singleton gestation with previous preterm birth and maternal short cervix
- Singleton gestation with previous spontaneous preterm birth (any cervical length)
- Singleton gestation with maternal short cervix, but no previous preterm birth

## Methods/design

### Protocol development and registration

This protocol has been registered in PROSPERO (CRD42017068299) and is reported in accordance with the Preferred Reporting Items for Systematic review and Meta-analysis Protocols (PRISMA-P) 2015 statement [17]. Representatives of all trials identified during project development (Table 1) have been invited to comment on and contribute to the development of this protocol. For transparency and to safeguard against perception of academic bias, a record is kept of trial investigator comment and the IPD-MA research team is responsible for making methodological decisions. Feedback on project design has also been obtained from consumer representatives.

### Inclusion and exclusion criteria

We aim to include all relevant trials irrespective of whether they are published or unpublished, where trials have been carried out, or which language they have been managed and reported in. We aim to include any trial that completed recruitment before July 2016 (allowing 1 year between completion and EPPPIC’s data collection for trial investigators to complete their own analyses). Such trials will be included in any future updates.

#### Population

Trials including asymptomatic women at increased risk of preterm birth including women who have experienced previous spontaneous preterm birth, a multiple gestation pregnancy, a short cervical length, or a positive fetal fibronectin test at randomization. Trials of progestogen given to women to prevent miscarriage will be excluded. Trials of progestogen administered for immediately threatened preterm birth including premature preterm rupture of membrane or uterine contractions will be excluded. Singleton, twin, and triplet pregnancies will be considered separately.

#### Intervention

Trials evaluating any form of progestogen are eligible, including natural progesterone and synthetic 17 alpha-hydroxyprogesterone caproate (17-OHPC), delivered by any route including vaginal gels, capsules and suppositories, intramuscular injection, intravenous injection, and oral administration. Different types of progestogen will be analysed separately. Planned and unplanned co-treatments are permitted provided that co-treatments are equally permitted on each intervention arm, or that trials with planned co-interventions make un-confounded comparison (e.g., progestogen + cerclage versus cerclage alone). Trials where administration of progestogen does not continue beyond the 16th week of pregnancy will be excluded.

#### Comparators

Trials that compare progestogen with placebo or with non-intervention will be included. Trials that compare progestogen with other active interventions such as cerclage will be excluded. Trials that compare different types of progestogen will be included in a secondary network meta-analysis exploring particularly the comparative effectiveness of vaginal progesterone, 17-OHPC, and oral dedroxyprogesterone acetate. A future project may extend to an IPD evaluation of all active interventions for prevention of preterm birth.

#### Outcomes

All trials that meet the above criteria will be included and contribute to the IPD-MA prespecified outcomes for which they collected data.

#### Study design

To limit potential for bias, only randomized controlled trials will be included. Quasi-randomized studies will be excluded. Cluster randomized and cross over trials will also be excluded.

### Trial identification

As is usual with IPD-meta-analyses, initial literature searches and eligibility screening have been carried out as part of protocol development. This is to ensure that a draft protocol can be sent to trial investigators along with their invitation to partner in the project, and is central to the collaborative approach. Inclusion criteria were established at the outset of the project.

Bibliographic searches of MEDLINE, Embase, CINAHL, and the Maternity and Infant Care databases as well as the Cochrane Pregnancy and Childbirth Review Group's specialized register were carried out during the development phase of the project (this is usual for IPD meta-analyses) and will be re-run at the end. An example MEDLINE search strategy is provided in Appendix A. Trial registers (ClinicalTrials.gov, ISCTRN, and the WHO ICTRP portal) were also searched to identify any unpublished and/or important ongoing trials. Unpublished trials that completed data collection before July 2016 were considered for inclusion. This cut-off was designed to allow trial investigators 1 year after recruitment to complete their own research and analyses. Authors of included trials have also been/will be asked to identify any unpublished trials of which they are aware.

Two researchers independently screened all titles and abstracts retrieved from electronic database and other searches. Full paper publications were then obtained for potentially relevant trials. Where no full paper existed and/or trial eligibility was uncertain, study authors were contacted and asked to provide further information.

Two researchers independently assessed the relevance of each trial using the fullest available information. Any discrepancies in screening decisions were resolved by consensus and discussion with a senior team member or clinician, as required.

‘Near miss’ studies that do not meet all of the inclusion criteria and have therefore been excluded from EPPPIC will be tabulated, and their bibliographic details listed with reasons for exclusion in the final EPPPIC report and PRISMA diagram.

### Data provision and coding

Trial investigators will be invited to supply data in a standardized format using standardized coding developed for EPPPIC. However, data will be accepted in any reasonable format and re-coded as necessary by the research team. Data will be requested for all women randomized, including any who were excluded from original trial analyses. Trial protocols and forms will also be collected. A list of data items to be collected is given in Appendix B.

Data supplied will have all names and identifying numbers removed. Individuals will either be labeled with numbers known only to the original trial team or numbered sequentially and trial investigators will be asked to keep a record of these numbers. This will enable any data queries to be traced back to the appropriate individual.

### Data storage and confidentiality

All IPD will be received via secure online transfer, or any other secure method such as secure FTP transfer or encrypted email. All data will be anonymous and held in a password-protected area of the Centre for Reviews and Dissemination's (CRD) server. No attempt will be made to re-identify participants and in the unlikely case of re-identification, confidentiality will be maintained. Access will be limited to staff working directly on the project. Copying data to laptop computers or memory sticks will be prohibited.

### Critical appraisal, data checking, and quality assurance

Critical appraisal and assessment of data quality will be based on trial protocols and publications and on IPD checking. Risk of bias will be assessed using the Cochrane risk-of-bias tool (RoB) [18]. Assessment will be undertaken by one researcher and if discrepant from RoB assessments reported in previous Cochrane [7] or other systematic reviews, will be checked independently by a second. Two researchers will independently assess trials not previously assessed and reported in a Cochrane or other good quality systematic review. Any disagreements will be discussed with a senior member of the EPPPIC IPD-MA research team.

All IPD will be checked on receipt. Data will be checked for internal consistency, baseline imbalance, and integrity of randomization. Patterns of missing data will be examined. Baseline data will be tabulated and compared with the trial publication and any inconsistencies noted. One researcher will run data checks, which will be independently checked by a second researcher. Findings of all data checking will be discussed with senior members of the research team.

Each individual trial will be analyzed (main outcomes only) and compared with corresponding published analyses (bearing in mind that there may be reasonable discrepancies, if, for example, previously excluded participants have been reinstated in the analyses, or additional follow up data have been provided). Any problems, uncertainties, or queries will be passed back to the responsible trial investigator for explanation and discussion.

Results of data checking may up- or down-weight implications of RoB assessments, for example, data checks may show that there is no evidence that risk of bias arising from the method of randomization has been realized. Any datasets that are judged to be of insufficient quality or completeness will be excluded from the analyses. This may be for the trial as a whole or for particular outcomes or analyses, depending on the nature of the problem.

### Data description

Descriptive tables and a narrative summary outlining the key design features and demographic characteristics of each included trial will be produced. Excluded trials will be listed with reasons for exclusion.

### Planned analyses

A detailed statistical analysis plan will be developed when the extent of available data is known, but before starting meta-analysis.

Analyses will evaluate overall effectiveness of main and additional outcomes on an intention to treat basis, that is, participants will be analyzed according to allocated treatment, irrespective of whether the treatment was received.

Three parallel but separate analyses focusing on (i) singleton and (ii) twin and (iii) triplet pregnancies will be undertaken. If there is no evidence of differences in effectiveness of progestogen in these three types of pregnancy, further exploratory analyses will incorporate all pregnancy types in a multi-level model. Twin and triplet pregnancies may be combined if there are insufficient triplet data for a viable analysis, and there is no clear evidence of differences between twin and triplet pregnancies. Separate analyses will be carried out for each type of progestogen. Further *exploratory analyses may* combine types of progestogen if there is no evidence of differences in effectiveness between them.

Maternal, pregnancy, neonatal, and longer-term developmental outcomes will be considered. A separate but complementary focus group study on views of women who have experienced preterm birth will identify outcomes that are especially important to pregnant women and their families, and which therefore lend themselves to shared decision-making.

Outcomes align broadly with the Core Outcomes in Women’s and Newborn health (CROWN) list [19]. Consideration of additional outcomes reflects the opportunity that IPD-MA provides to leverage maximum information from preexisting data. As many outcomes are being explored, findings will be interpreted with the knowledge that there is a high likelihood that statistically significant results will arise by chance. Findings will therefore be interpreted cautiously and in the round, with biological plausibility and consistency across related outcomes and across trials lending credence; and inconsistency suggesting that results may be spurious and should be interpreted conservatively.

A final list of outcomes to be addressed will depend on what variables trials have collected and are available from the included trials. Outcomes with low numbers of events may be combined into more general categories for formal analysis. Appendix B lists the data items that will be requested, including baseline and outcome variables.

#### Main outcomes

- **Preterm birth or fetal death** (< =37, <= 34, <= 28 weeks)
- **Serious neonatal complications or fetal/infant death**
  (broncho-pulmonary dysplasia, severe intraventricular hemorrhage, nectrotizing enterocolitis, confirmed sepsis, patent ductus arteriosus, retinopathy of prematurity)
- **Neurosensory disability** (measured at 18 months or later) or **infant/child death**
  (cerebral palsy, visual impairment, hearing impairment, epilepsy, intellectual impairment, developmental delay)
- **Important maternal morbidity or maternal death**
  (gestational diabetes, gestational hypertension, preeclampsia, maternal infection)
- **Fetal/infant death**
  (fetal death occurring at any point after trial entry, stillbirth, or death of live born infant before hospital discharge following birth, or to 28 days, whichever is longer)

With component outcomes defined as follows

#### Additional outcomes

- Gestational age at birth
- Prolongation of pregnancy (interval between randomization and delivery)
- Prelabor spontaneous rupture of membranes (≤28, ≤34, ≤37 weeks and >37 weeks)
- Non-spontaneous onset of labor (medically induced or caesarian before labor)
- Mode of birth (vaginal/caesarian birth)
- Gestational diabetes
- Preeclampsia
- Gestational hypertension
- Maternal infection
  (chorioamnionitis during labor; intrapartum fever, postpartum fever requiring antibiotics)
- Adverse/side effects of treatment
- Maternal death
- Fetal death/stillbirth (death of fetus after randomization)
- Death of newborn (death of live born infant to hospital discharge following birth)
- Birth weight (adjusted for gestational age and sex)
- Admission to neonatal intensive or special care unit (NICU/SCU)
- Length of stay in NICU/SCU
- Respiratory distress syndrome (as defined in the trial)
- Use of respiratory support (mechanical, CPAP, high flow nasal cannula)
- Bronchopulmonary dysplasia (as defined in trial)
- Periventricular leukomalacia
- Necrotizing enterocolitis (grade II or III)
- Neonatal infection (antenatal, early, late; as defined in trial)
- Confirmed neonatal sepsis
- Patent ductus arteriosus (treated for)
- Severe intraventricular hemorrhage (grade III or IV)
- Retinopathy of prematurity (stage 3 or worse)
- Major congenital anomalies including cardiac malformation
- Death and cause of death after discharge (from hospital following birth)
- Cerebral palsy
- Visual impairment (visual acuity worse than 6/60, 20/200 in better eye)
- Hearing impairment (requiring amplification or worse)
- Epilepsy
- Intellectual impairment (as defined in trial)
- Developmental delay (mild, moderate, or severe on Bailey scale or equivalent)
- Growth outcomes (including height, weight, head circumference, if possible with reference to appropriate growth chart centiles)

Neurosensory disabilities will be collected individually, but it is anticipated that events will be few and that they will need to be combined for analysis.

We will also analyze an exploratory composite outcome of birth after 37 weeks of gestation of a surviving baby with no serious neonatal complication or neurosensory disability and with a surviving mother with no long-term adverse events.

#### Analysis of potential effect modifiers

Potential effect modifiers will be investigated to explore whether any particular therapeutic approaches are more effective than others, and/or whether there are particular types of women who derive greater benefit (or harm) from intervention.

Trial-level intervention characteristics

- Intended dose (separately for each type of progestogen)
- Intended early co-intervention(s)

Participant-level maternal and pregnancy characteristics at trial entry

- Previous spontaneous preterm birth
- Gestational age at previous preterm birth
- Cervical length (continuous variable and using thresholds ≤15 mm, ≤20 mm, ≤25 mm, ≤30)
- Positive fetal fibronectin test
  (continuous variable and using thresholds ≥ 10 ng/ml, ≥ 50 ng/ml, ≥ 200 ng/ml)
- Singleton or multiple gestation pregnancy
  (singleton/multiple and singleton/twin/triplet gestation pregnancy)
- Chorionicity and amnionicity
- Gestational age at randomization/initiation of treatment
- Maternal age
- Ethnicity (within comparable geographical locations)
- Assisted conception
- Chronic hypertension
- Prepregnancy diabetes
- Smoking during pregnancy
- Maternal body mass index (categorized using WHO definitions)

### Sensitivity and supplementary analyses

Sensitivity analyses will assess the impact of trial design features and alternative approaches to synthesis. In addition to those listed, further analyses may be undertaken where principle analyses suggest that further investigation may be informative. All analyses will be described according to whether they were principle or sensitivity analyses and whether they were preplanned or ad hoc.

- Supplemented (where possible) with aggregate data from published reports of trials for which IPD is not available
- Restricted to trials at low risk of bias with respect to design features such as blinding
- Restricted to trials that have been prospectively registered
- Analysis of cervical length restricted to trials that used ultrasound measurement
- Separate analyses of trials measuring developmental outcomes directly and those that used questionnaires
- Separate analyses of trials measuring intellectual impairment based on health records and those that used parent reports
- Multivariable analysis including singleton and multiple gestation pregnancies
- Analysis of ethnicity across all trial locations
- Analyses of covariate treatment interaction for additional outcomes where analyses of main outcomes suggests that further analysis may be informative

### Statistical methods

#### Outcome measures

Dichotomous outcomes will be analyzed by calculating the risk ratio for the effect of progestogen compared to the control treatment (placebo or usual care). Odds ratios may be used where risk ratios cannot be computed. For continuous outcomes mean differences between treatment arms will be reported. Hazard ratios will be calculated for time-to-event outcomes.

#### Unit of analysis

Maternal and birth outcomes will use the pregnancy as the unit of analysis. Infant outcomes will use the baby as the unit of analysis. Because infants within a multiple pregnancy are more similar to each other than to babies in other pregnancies (statistically, they are not independent), analyses will be adjusted for this clustering (provided that the additional complexity does not make one-stage models computationally intractable) [20].

#### One- and two-stage models

The IPD will be synthesized across trials using meta-analysis. Both ‘two-stage’ models (where effect estimates are calculated for each trial, and subsequently pooled in a meta-analysis) and ‘one-stage’ models (where all IPD from all trials are analyzed in one step, while accounting for the clustering of participants within trials) will be used.

#### Two-stage models

Two stage models will be used as an initial analysis for all the main outcomes listed previously. Effect estimates (relative risk (RR), mean difference (MD), or hazard ratio (HR)) will be estimated for each trial, and then combined using random effects meta-analysis. This will generate forest plots enabling results across trials to be compared visually, heterogeneity investigated and differences across subgroups visualized. All of these aspects will be essential in gaining a full understanding of the underlying dataset, and will motivate the choice of more complex one-stage models [21]. Heterogeneity will be quantified using the *I* ^2^ statistic.

#### One-stage models

One-stage models will be fitted for all outcomes. One-stage analyses will pool IPD from all trials using a generalized linear mixed model framework, which accounts for potential heterogeneity across trials. For continuous outcomes, linear mixed-effect models will be fitted. For dichotomous outcomes, logistic mixed-effect models will be used to calculate relative risks or odds ratios where relative risks are computationally intractable.

#### Impact of trial and participant characteristics

The impact of the trial and patient-level characteristics on treatment effect (that is, treatment–covariate interactions) will be examined. For trial-level covariates, the trials will be divided into groups according to the characteristic and meta-analyses performed within each subgroup. The more formal analysis of interactions will use one-stage models, where treatment covariate interactions will be added to existing one-stage models for treatment effect. This will enable us to take account of multiple participant characteristics when comparing progestogen with placebo or usual care (stratified by trial), and will also enable exploration of potential treatment interactions in a multivariable way. Models will be compared in terms of goodness of fit and parsimony using the Akaike information Criterion (AIC).

#### Time to event analyses

For the analyses of whether progestogen prolongs pregnancy, the hazard ratio for the effect of progesterone will be calculated within each trial by fitting a Cox proportional hazards model. Tests for proportional hazards will be performed. Hazard ratios will be pooled across trials using random effects meta-analysis. If the proportional hazards assumption is reasonable, one-stage random effects Cox models will be fitted. Treatment–covariate interactions will be included in these models as required.

#### Relative and absolute differences

Absolute differences will be calculated by applying the resulting risk ratios or hazard ratios to appropriate baseline incidences (calculated from suitable meta-analyses across the trial control arms). Numbers needed to treat and numbers needed to harm will similarly be calculated for a range of plausible baseline measures.

### Unavailable trials and missing data

Every effort will be made to minimize the amount of missing data, including requesting information for any randomized participants that were excluded from the original trial analyses. Where IPD cannot be obtained for a trial, and where possible, aggregate data will be extracted from publications and combined with the IPD-MA results in a sensitivity analysis. Where covariate data are missing for some participants, a complete case analysis will be used in the first instance (i.e., excluding patients with missing data). If there are substantial missing data (around 10% for any outcome or covariate), multiple imputation within each trial will be used to impute missing covariates, where this is computationally feasible. Trials that have not recorded particular outcomes or particular covariates will not contribute to those analyses. Sensitivity analyses based on best and worst case scenarios will be used to assess the impact of missing outcome data.

### Network meta-analysis

If sufficient suitable data are available, a network meta-analysis will compare all types of progesterone and routes of administration. This will incorporate direct evidence from head to head trials and indirect evidence from trials comparing each type of progestogen with no intervention. Analyses will be conducted for the main outcomes listed earlier.

Two statistical models will be used: first, the Bayesian models of Lu and Ades [22], which are the most commonly used methods for network meta-analysis. The one-stage meta-analysis models described above will also be extended to include multiple treatment arms. The results of the two approaches will be compared. Both approaches will use random effects to account for heterogeneity. Potential network inconsistency will be investigated by comparing results to results from direct pairwise meta-analyses. If there is evidence of differences, node-splitting models will be used to investigate inconsistency further.

### Software

All analyses will be performed at CRD using the R software package [23]. Two-stage analyses will additionally use the meta and metafor libraries [24, 25]. One-stage models will be fitted via the lme4 library, and one-stage Cox models will use the coxme library. Forest plots will be produced using in-house R code. For the network meta-analysis, WinBugs and the GeMTC package in R will be used. https://www.mrc-bsu.cam.ac.uk/software/bugs/the-bugs-project-winbugs/

### Reporting

Results will be presented and discussed at a dedicated meeting of the EPPPIC group. Discussion will inform the interpretation of results and development of the final report. An audited list of comments received, and actions taken will be maintained. The results of the IPD-MA will be reported in accordance with PRISMA-IPD [26]. Authorship of the final journal publication will be by the EPPPIC group. Plain language summaries of findings will be produced.

### Data repository

Our aspiration is that at the conclusion of our analyses, data will be archived in a repository that will be developed and curated under the aegis of the Cochrane Pregnancy and Childbirth Group. This would establish the nucleus of a data repository that can grow over time and underpin future IPD syntheses and other research projects in pregnancy and childbirth topics. Trial investigators will be given the opportunity to elect to share their data supplied to EPPPIC with the repository under a range of options that the repository will offer. No further work would be required to share data in this way. Participation in the repository is optional. Trialists who participate in the EPPPIC group and IPD-MA are not required to contribute their data to the repository.

## Discussion

The EPPPIC individual participant data meta-analysis is an international collaborative project that will be carried out on behalf of, and published by, the EPPPIC group. All trial investigators who share data will be active participating members of this group. The project is endorsed and advised by the EPPPIC secretariat, which includes trial investigators responsible for the largest trials, representation from Cochrane, the US Patient-Centered Outcomes Research Institute (PCORI), the National Institute for Health Research (NIHR) in the UK, the March of Dimes (US), and consumer stakeholders.

EPPPIC will pay attention to the views of consumer representatives and of women at risk of preterm birth (obtained in the separate but linked focus group project), and will provide important data and tailored outputs to inform shared decision-making and a stratified and more personalized approach to intervention.

Initial results will be presented to all EPPPIC members at a dedicated meeting planned for early 2018, with associated discussion informing the development of a final report and manuscript. The collaborative aspects of the work and involvement of stakeholders and those responsible for the included trials may help reach consensus, and will be an important facet of knowledge mobilization activity.

**Table 1:** Provisional list of trials potentially eligible for inclusion in EPPPIC (progestogen versus placebo/usual care). This table will be updated as new trials are identified. Publication details for individual trials are available on request

EPPPIC identifier	Trial setting	Recruitment years	Progestogen type, dose, intervention, period Comparator	Main indication(s) Gestation at randomization	Singleton/multiple pregnancy	*N*
A: Eligible trials comparing progestogen with standard care or placebo^a^						
1	Single center Egypt (Cairo)	2008–2009	Vaginal Micronized progesterone 400 mg suppository daily 18–24 to 37 weeks Placebo	First pregnancy by IVF/ICSI 18–24 weeks	SingletonTwin	313
2	Single center Iran (Khorramabad)	–	Vaginal Progesterone 100 mg daily 24 to 34 weeks Standard care	Previous SPTBProphylactic cerclage Uterine anomalies Gestation unclear	Singleton	150
3	Single center Lebanon (Beirut)	2006–2012	Intramuscular injection 17-OHPC 250 mg weekly 16–20 to 36 weeks Castor oil	Twin pregnancy 12–20 weeks	Twin	293
4	Multi center USA	2004–2006	Intramuscular injection 17-OHPC 250 mg weekly 16–20 to 35 weeks Castor oil	Triplet pregnancy 16–20 weeks	Triplet	134
5	Single center Turkey (Istanbul)	2004–2007	Vaginal Progesterone 100 mg suppository daily 24 to 34 weeks Placebo	Twin pregnancy Previous SPTB Uterine anomalies 24 weeks	Singleton Twin	160
6	Multi center USA	2004–2008	Intramuscular injection 17-OHPC 250 mg weekly 16–24 to 34 weeks Castor oil	Trichorionic-triamniotic triplet pregnancy 15–23 weeks	Triplet	81
7	Multi center USA	2004–2009	Intramuscular injection 17-OHPC 250 mg weekly 16–24 to 34 weeks Castor oil	Dichorionic-diamniotic twin pregnancy 15–23 weeks	Twin	240
8	Multi center Australia	2005–2009	Vaginal Progesterone 100 mg suppository nightly 20–24 to 34 weeks Placebo	SPTB preceding pregnancy 18–24 weeks	Singleton	787
9	Single center Brazil (São Paulo)	1996–2001	Vaginal Progesterone 100 mg suppository nightly 24 to 34 weeks Placebo	Previous SPTBPrevious cerclage Uterine anomalies Gestation not reported	Singleton	157
10	Single center Egypt (Cairo)	–	Vaginal Progesterone 200 mg daily 24 to 34 weeks Placebo	Twin pregnancy	Twin	100
11	Multi center International	2003–2006	Vaginal Progesterone 200 mg suppository nightly 24–25 to 34 weeks Safflower oil	Short cervix (≤15 mm) 20–25 weeks	Singleton twin	250
12	Single center Pilot USA (Ohio)	2006–2009	Oral Micronized progesterone 2 × 200 mg capsule daily 16–20 to 34 weeks Placebo	Previous live SPTB < 20 weeks	Singleton	^b^33
13	Multi center USA	2007–2011	Intramuscular injection 17-OHPC 250 mg weekly 16–23 to 37 weeks Castor oil	Nulliparous Short cervix (< 30 mm) 16–23 weeks	Singleton	657
14	Single center Finland (Oulu)	–	Intramuscular injection 17-OHPC 250 mg weekly 28–33 to 37 weeks Placebo	Twin pregnancy 28–33 weeks	Twin	77
15	Multi center International	2008–2010	Vaginal Progesterone 90 mg gel daily 20–24 to 37 weeks Placebo	Short cervix (10–20 mm) 19–24 weeks	Singleton	465
16	Single center Egypt (Cairo)	2006–2008	Intramuscular injection 17-OHPC 250 mg weekly Second trimester to 36 weeks Saline	Previous SPTB Second trimester	Singleton	50
17	Single center USA (Baltimore)	–	Intramuscular injection 17-OHPC 250 mg weekly Unclear to 37 weeks Castor oil + benzyl benzoate	2 previous SPTB 2 SAs or (1 SPTB and 1 SA) in immediately preceding pregnancy < 24 weeks	Singleton Twin	50
18	Multi center Netherlands	2006–2009	Intramuscular injection 17-OHPC 250 mg weekly 16–20 to 36 weeks Castor oil	Multiple pregnancy 15–19 weeks	Twin Triplet Quadruplet	671
19	Single center India (Chandigarh)	2004–2006	Vaginal Micronized progesterone 100 mg capsule nightly 20–24 to 36 weeks Standard care	Previous singleton SPTB 16–24 weeks	Singleton	100
20	Multi center USA	1999–2002	Intramuscular injection 17-OHPC 250 mg weekly 16–20 to 36 weeks Castor oil	Previous SPTB 15–21 weeks	Singleton	463
21	Single center Albania (Tirana)	–	Intramuscular injection 17-OHPC Dose not reported, daily 15–22 to 34 weeks Oral Progesterone daily (dose not reported, daily 15–22 to 34 weeks Placebo	High risk of SPTB 15–22 weeks	–	121
22	Multi center UK	2004–2008	Vaginal Progesterone 90 mg gel daily 24 to 34 weeks Placebo	Twin pregnancy 22 weeks	Twin	500
23	Multi center UK	2009–2013	Vaginal Progesterone 200 mg suppository nightly 22–24 to 34 weeks Placebo	Previous SPTB Short cervix (≤ 25 mm) Positive FFT and other risk factors for SPTB	Singleton	1228
24	Multi center USA	2004–2007	Vaginal Progesterone 90 mg gel daily 18–23 to 37 weeks Vaginal moisturizer	Singleton SPTB in most recent pregnancy 16–23 weeks	Singleton	659
25	Single center Iran (Tehran)		Intramuscular injection 17-OHPC 250 mg weekly 16–20 to 34 weeks Placebo	Women ≥ 35 years	–	260
26	Single center India (Delhi)	2005–2006	Oral Micronized progesterone 100 mg capsule twice daily 18–24 to 36 weeks Placebo	Previous SPTB 18–24 weeks	Singleton	150
27	Multi center Denmark and Austria	2006–2008	Vaginal Micronized progesterone 200 mg suppository daily 20–24 to 34 weeks Safflower oil, gelatine, glycerol, titanium dioxide (E171)	Diamniotic twin pregnancy Chorionicity <16 weeks 18–24 weeks	Multiple	677
28	Multi center USA	2004–2006	Intramuscular injection 17-OHPC 250 mg weekly 16–20 to 35 weeks Castor oil	Twin pregnancy 16–20 weeks	Twin	661
29	Single center Iran (Mashhad)	2007–2008	Intramuscular injection 17-OHPC 250 mg weekly 16 to 37 weeks Standard care	Previous SPTB Not reported	Singleton	100
30	Multi center France	2006–2010	Intramuscular injection 17-OHPC 500 mg twice weekly 24–32 to 36 weeks Standard care	Dichorionic diamniotic twin pregnancy Cervix ≤25 mm 24–32 weeks	Twin	165
31	Multi center Spain	2006–2008	Vaginal Progesterone (1) 2 × 200 mg suppository nightly (2) 1 × 200 mg + 1 × placebo suppository nightly 20 to 34 weeks (3) 2 x placebo suppository nighly Peanut oil + soy lecithin	Dichorionic diamniotic twin pregnancy 20 weeks	Twin	294
32	Two center Canada (Calgary)	2006–2010	Vaginal Progesterone 90 mg gel daily 16–21 to 36 weeks Placebo	Multiple gestation 16–21 weeks	Twin Triplet	84
33	Single center Brazil (São Paulo)	2007–2013	Vaginal Progesterone 200 mg suppository nightly 18–21 to 34 weeks Placebo	Naturally conceived diamniotic twin pregnancy 18–21 weeks	Twin	390
34	Single center Iran (Tehran)	2010–2012	Vaginal Progesterone 400 mg suppository nightly 16–22 to 36 weeks Placebo	Previous SPTB Cervix ≤ 28 mm + cerclage Uterine anomalies Uterine intramural myoma ≥ 7 cm 16–22 weeks	Singleton	103
35	Two center Egypt (Mansoura)	2012–2014	Vaginal Progesterone 400 mg suppository daily 20–24 to 37 weeks Standard care	Dichorionic twin pregnancy Cervix 20–25 mm 20–24 weeks	Twin	250
36	Multi center Netherlands	2009–2013	Vaginal Micronized progesterone 200 mg suppository daily 22 to 34 weeks Placebo	Nulliparous Multiparous without SPTB < 34 weeks gestation Cervix ≤30 mm 18–22 weeks	Singleton	80
37	Single center India (Shimla)	–	Vaginal Progesterone 100 mg suppository 24–28 to 34 weeks Placebo	Previous SPTB 24–28 weeks	Singleton	80
38	Single center Pakistan (Bahalwapur)	2011	Intramuscular injection 17P 250 mg weekly 16–20 to 36 weeks Placebo	Previous SPTB 16–20 weeks	Singleton	60
45	Single center USA (Jackson)	–	Intramuscular injection 17-OHCP 250mg weekly Up to 34 weeks or delivery Castor oil	Twin pregnancy 20–30 weeks	Twin	30
B: Eligible trials comparing different types of progestogen						
21	Single center Albania (Tirana)	–	Intramuscular injection 17-OHPC Dose not reported, daily 15–22 to 34 weeks Oral Progesterone daily (dose Dose not reported, daily 15–22 to 34 weeks Placebo	High risk of SPTB 15–22 weeks	–	121
39	Single center Egypt (Tanta)	2010–2011	Vaginal Micronized progesterone 200 mg suppository daily 20–24 to 34 weeks Intramuscular injection Progesterone 100 mg every 3rd day 20–24 to 34 weeks	Previous SPTB Cervix ≥ 15 mm 20–24 weeks	Singleton	160
40	Single center Iran (Yazd)	2012–2015	Vaginal Progesterone 200 mg suppository daily 16–20 to delivery Intramuscular injection 17-OHPC 250 mg weekly 16–20 weeks to delivery	Previous SPTB or Cervix < 25 mm (not both) 16–20 weeks	Singleton	78
41	Single center Saudi Arabia (Khamis Mushayt)	2009–2011	Vaginal Micronized progesterone 90 mg gel daily 14–18 to 36 weeks Intramuscular injection 17-OHPC 250 mg weekly 14–18 to 36 weeks	Previous mid-trimester SPTB Previous cerclage 14–18 weeks	Singleton	518
42	Single center Iran (Tehran)	2012–2015	Vaginal Progesterone 400 mg suppository daily 16–24 to 36 weeks 17-OHPC 250 mg weekly 16–24 to 36 weeks	Cervix < 25 mm 16–24 weeks	Singleton	304
43	Single center USA (Oklahoma)	2007–2010	Intramuscular injection 17-OHPC 250 mg weekly 16–21 to 37 weeks Vaginal Micronized progesterone 100 mg suppository daily 16–21 to 37 weeks	Previous live singleton SPTB 16–21 weeks	Singleton	174
44	Single center Iran (Yazd)	2010–2011	Intramuscular injection 17-OHPC 250 mg weekly 16 to 36 weeks Placebo	Assisted conception 16 weeks	Singleton	106

^a^Eligibility as of 15/07/2017, eligibility of all trials subject to final confirmation. Abbreviations used: *IVF/ICSI* in vitro fertilization/intracytoplasmic sperm injection, *N* number randomized, *SPTB* spontaneous preterm birth, *IMI* intramuscular injection, *SA* spontaneous abortion, *FFT* fetal fibronectin test

^b^Analyzed, not randomized (number randomized unclear)

## Appendix A

### Search strategy

Database: Ovid MEDLINE(R) Epub Ahead of Print, In-Process and Other Non-Indexed Citations, Ovid MEDLINE(R) Daily and Ovid MEDLINE(R) (1946 to present).

Search strategy:

1. exp. Progesterone/ (68227)
2. progesterone$.mp. (103366)
3. hydroxyprogesterones/ or 17-alpha-hydroxyprogesterone/ (4868)
4. 17-hydroxyprogesterone caproate.mp. (63)
5. 17-OHPC.mp. (48)
6. 17OHPC.mp. (18)
7. 17Pc.mp. (11)
8. progestins/ or 20-alpha-dihydroprogesterone/ or algestone/ or algestone acetophenide/ or allylestrenol/ or desogestrel/ or dydrogesterone/ or flurogestone acetate/ or gestrinone/ (12661)
9. 20 alpha dihydroprogesterone.mp. (646)
10. 20-alpha-dihydroprogesterone.mp. (646)
11. 20 alpha-dihydroprogesterone.mp. (646)
12. 20-alpha dihydroprogesterone.mp. (646)
13. algestone.mp. (171)
14. allylestrenol.mp. (154)
15. desogestrel.mp. (1802)
16. dydrogesterone.mp. (603)
17. flurogestone acetate.mp. (115)
18. gestrinone.mp. (264)
19. progestogen$.mp. (5291)
20. progestative$.mp. (248)
21. medroxyprogesterone acetate.mp. (7020)
22. 1 or 2 or 3 or 4 or 5 or 6 or 7 or 8 or 9 or 10 or 11 or 12 or 13 or 14 or 15 or 16 or 17 or 18 or 19 or 20 or 21 (123754)
23. Premature Birth/ (10507)
24. Obstetric Labor, Premature/ (13369)
25. premature birth$.mp. (13486)
26. preterm birth$.mp. (13895)
27. pre-term birth$.mp. (356)
28. pre term birth$.mp. (356)
29. PTB.mp. (4521)
30. preterm labor$.mp. (5634)
31. pre-term labor$.mp. (97)
32. pre term labor$.mp. (97)
33. preterm labor$.mp. (1729)
34. pre-term labor$.mp. (134)
35. pre term labor$.mp. (134)
36. PTL.mp. (947)
37. premature labor$.mp. (2314)
38. premature labor$.mp. (793)
39. preterm rupture$.mp. (422)
40. pre-term rupture$.mp. (8)
41. pre term rupture$.mp. (8)
42. premature rupture$.mp. (7345)
43. preterm deliver$.mp. (9824)
44. pre-term deliver$.mp. (406)
45. pre term deliver$.mp. (406)
46. premature deliver$.mp. (2897)
47. (perinatal adj3 (outcome$ or morbidit$ or mortalit$)).ti,ab. (18841)
48. (neonatal adj3 (outcome$ or morbidit$ or mortalit$)).ti,ab. (19966)
49. (pregnancy adj3 (outcome$ or morbidit$ or mortalit$)).ti,ab. (26850)
50. (maternal adj3 (outcome$ or morbidit$ or mortalit$)).ti,ab. (23799)
51. (developmental adj3 (outcome$ or morbidit$ or mortalit$)).ti,ab. (3796)
52. 23 or 24 or 25 or 26 or 27 or 28 or 29 or 30 or 31 or 32 or 33 or 34 or 35 or 36 or 37 or 38 or 39 or 40 or 41 or 42 or 43 or 44 or 45 or 46 or 47 or 48 or 49 or 50 or 51 (114515)
53. 22 and 52 (2300)
54. randomized controlled trial.pt. (469461)
55. controlled clinical trial.pt. (94440)
56. randomized.ab. (411777)
57. placebo.ab. (191551)
58. drug therapy.fs. (2019667)
59. randomly.ab. (285289)
60. trial.ab. (431708)
61. groups.ab. (1756447)
62. 54 or 55 or 56 or 57 or 58 or 59 or 60 or 61 (4164393)
63. exp. animals/ not humans.sh. (4440485)
64. 62 not 63 (3601842)
65. 53 and 64 (820)

## Appendix B

### Data items to be collected

Trial level data items to be collected

- Trial registration number, if applicable
- Method of randomization
- Date trial started
- Date trial closed
- Control arm details
- For each treatment arm
- Type of progestogen (17-OHPC/medroxyprogesterone acetate/natural)
- Route of administration (intramuscular/iv/vaginal/oral)
- Dose (intended progestogen dose (mg) and number of doses per week)
- Details of planned co-interventions/intervention policy
- Method of measuring cervical length
- Method of calculating gestational age/due date
- Scale used to measure developmental delay
- Method of assessing intellectual impairment
- Staging and grading systems used

Individual-level data items to be collected

Baseline data at or close to randomization and maternal/pregnancy outcomes

- Maternal unique ID (does not include participant name or identifier)
- Date of randomization
- Gestational age at randomization
- Maternal age at randomization
- Ethnicity
- Assisted conception
- Parity
- Prior history of spontaneous preterm birth
- Gestational age at most recent previous preterm birth
- Reason for most recent preterm birth
  (preterm labor, preterm prelabor rupture of the membranes, antepartum hemorrhage, preeclampsia, other)
- Singleton/twin/triplet gestation pregnancy
- Prerandomization death in utero
- Chorionicity and amnionicity (multiple gestation only)
- Cervical length
- Fetal fibronectin level
- Chronic (prepregnancy) diabetes
- Chronic hypertension
- Smoking during pregnancy
- Maternal BMI at randomization
- Treatment arm assigned
- Gestation at start and end of treatment
- Discontinuation of study treatment
- Co-treatment(s) received when asymptomatic
- Late use of antibiotics
- Late use of tocolytics
- Late use of corticosteroids
- Preeclampsia
- Prelabor spontaneous rupture of membranes and gestational age at occurrence
- Labor onset
- Gestational age at birth
- Gestational diabetes mellitus
- Gestational hypertension
- Gestational hypertension
- Chorioamnionitis
- Intrapartum fever
- Post-partum fever
- Maternal death and gestation at which maternal death occurs
- Adverse effects (vaginal irritation, itching, discharge, discomfort; nausea; vomiting; hot flushes; depression; headache; joint or pubic pain; swelling; rash, bruising, itching at injection site; unspecified/other)
- Serious adverse events, SUSARs
- Whether woman excluded from trial analysis and reason for exclusion

For each baby born

- Maternal unique ID
- Baby ID
- Mode of birth
- Live or still born
- Estimated gestational age at death of still born
- Sex
- Birth weight
- Length and head circumference
- Admission to NICU/SCU, length of stay
- Age at discharge from hospital
- Assisted ventilation and duration
- Treated for retinopathy of prematurity
- Respiratory distress syndrome
- Treated for bronchopulmonary dysplasia
- Infection and type (antenatal, early onset, late onset)
- Treated for necrotizing enterocolitis
- Confirmed neonatal sepsis
- Intraventricular hemorrhage
- Treated for patent ductus arteriosus
- Teratogenic effects/major anomalies
- Death of live born before hospital discharge and age at death
- Cause of death
- Whether new born excluded from trial analysis and reason for exclusion

Where long-term developmental outcomes have been collected

- Baby ID
- Cerebral palsy, severity (mild, moderate, severe)
- Visual impairment (acuity worse that 6/60 in better eye or as defined in trial)
- Hearing impairment requiring amplification or worse
- Epilepsy
- Intellectual impairment (as defined in trial)
- Developmental delay (mild/moderate/severe, Bailey scale or equivalent)
- Growth outcomes (height, weight, head circumference) and age at measurement
- Death of child and age at death and cause of death
- Whether child excluded from trial analysis and reason for exclusion
